# Synovial Fluid Markers and Extracellular Vesicles in Rheumatoid Arthritis

**DOI:** 10.3390/medicina60121945

**Published:** 2024-11-26

**Authors:** Veronika Smolinska, Daniela Klimova, Lubos Danisovic, Stefan Harsanyi

**Affiliations:** 1 Institute of Medical Biology, Genetics and Clinical Genetics, Faculty of Medicine, Comenius University in Bratislava, Sasinkova 4, 811 08 Bratislava, Slovakia; smolinska7@uniba.sk (V.S.); daniela.klimova@fmed.uniba.sk (D.K.); lubos.danisovic@fmed.uniba.sk (L.D.); 2 National Institute of Rheumatic Diseases, Nábrežie Ivana Krasku 4, 921 12 Piestany, Slovakia

**Keywords:** rheumatoid arthritis, synovial fluid, extracellular vesicles, exosomes, biomarkers

## Abstract

In recent years, numerous potential prognostic biomarkers for rheumatoid arthritis (RA) have been investigated. Despite these advancements, clinical practice primarily relies on autoantibody tests—for rheumatoid factor (RF) and anti-citrullinated protein antibody (anti-CCP)—alongside inflammatory markers, such as the erythrocyte sedimentation rate (ESR) and C-reactive protein (CRP). Expanding the repertoire of diagnostic and therapeutic biomarkers is critical for improving clinical outcomes in RA. Emerging evidence highlights the significance of synovial fluid biomarkers, including aggrecan, matrix metalloproteinases, glucosyl-galactosyl-pyridinoline, hyaluronic acid, S100 proteins, calprotectin, and various cytokines, as well as immunological markers. Additionally, specific components of extracellular vesicles, such as non-coding RNAs, heat shock proteins, and lipids, are gaining attention. This review focuses on molecular markers found in synovial fluid and extracellular vesicles, excluding clinical and imaging biomarkers, and explores their potential applications in the diagnosis and management of RA.

## 1. Introduction

Rheumatoid arthritis (RA) is a complex, chronic autoimmune disorder that primarily targets the synovial joints but can also impact multiple organs. It progresses in distinct phases, beginning with an asymptomatic genetic risk phase, moving to a preclinical stage with detectable biomarkers such as rheumatoid factor (RF) and anti-citrullinated protein antibodies (ACPAs)—mostly anti-cyclic citrullinated peptides (anti-CCPs)—and culminating in a clinical phase where joint inflammation and symptoms become apparent. The disease manifests with pain, stiffness, swelling, and loss of joint function, which can lead to deformity and disability if untreated. Systemic symptoms, including fatigue, fever, and weight loss, may also occur, and some individuals may develop complications involving the heart, lungs, and blood vessels [1]. Early and effective intervention is essential, as it can significantly improve patient outcomes by reducing irreversible joint damage and enhancing quality of life [2]. The identification of biomarkers that can differentiate aggressive, fast-progressing RA from milder forms is becoming more and more important in clinical practice. Such biomarkers enable clinicians to initiate targeted, intensive treatment plans promptly, optimizing patient prognosis by preventing long-term complications [3]. A systematic review reported the highest mean point prevalence in North America at 1.46%, followed by Europe at 0.53% [4].

Research in the field of biological and targeted synthetic disease-modifying anti-rheumatic drugs (DMARDs) gradually increases the number of available treatments. Methotrexate, a conventional synthetic DMARD, remains the first-line treatment for most patients due to its effectiveness and familiarity in clinical settings. However, biological and targeted synthetic DMARDs, such as TNF and Janus kinase (JAK) inhibitors, have expanded therapeutic options and are increasingly used for patients with inadequate responses to traditional therapies [5]. Although these newer agents offer potent anti-inflammatory effects and can halt disease progression, they also vary widely in cost, toxicity, and patient-specific efficacy. This variability underscores the importance of robust prognostic biomarkers to guide treatment decisions, helping clinicians to balance the intensity of therapy with potential risks, particularly in early-stage RA [6].

Biomarkers play a crucial role in clinical practice by providing objective measurements that reflect biological conditions. They can be utilized to evaluate risk, confirm the presence of a disease (diagnostic), monitor the progression of a disease (prognostic), and assess both the response (predictive) and the intensity of response (pharmacodynamic) to treatment. A significant challenge in rheumatology is the difficulty in forecasting long-term outcomes for patients exhibiting early symptoms indicative of arthritis [7]. Increasing evidence suggests that implementing aggressive treatments during the early stages of arthritis is vital for reducing radiographic damage and preventing disability. The early detection of easily measurable and predictive biomarkers is essential for categorizing arthritis patients according to their disease trajectory and mitigating the risks associated with over- or undertreatment. The current biochemical assays that measure RF, an autoantibody commonly used in RA diagnosis, demonstrate a sensitivity of only approximately 63%. Research is urgently needed to discover reliable biomarkers during the pre-symptomatic and initial clinical phases of arthritis to facilitate timely and effective disease management [8,9].

This review examines the latest advancements concerning RA biomarkers sourced from synovial fluid, emphasizing their potential for diagnostic applications.

## 2. Synovial Fluid Markers

**Figure 1:**
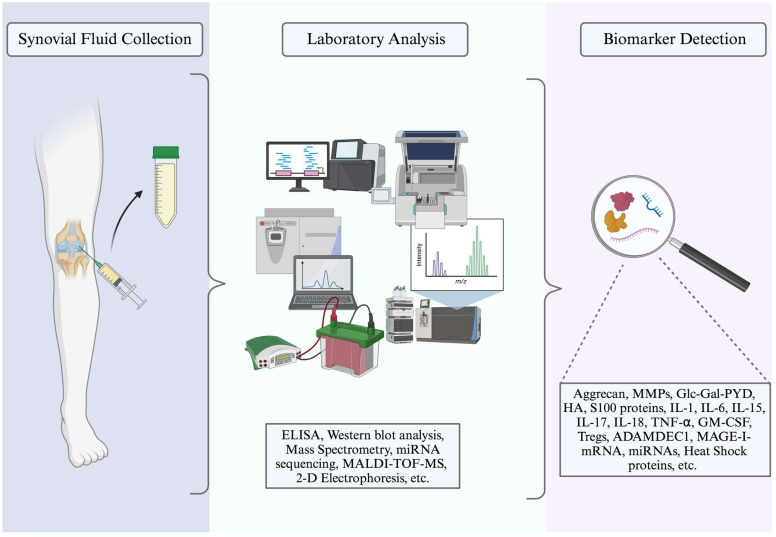
Synovial fluid collection, laboratory analysis, and biomarker detection.

Understanding key joint components—synovial fluid (SF), synovial membrane, and articular cartilage—is essential to study joint diseases effectively. The synovial membrane, made of macrophages and synovial fibroblasts in a collagen-rich matrix, regulates exchanges with nearby blood capillaries. Articular cartilage, composed of chondrocytes within a collagen-proteoglycan matrix, is critical for joint function. SF, a hyaluronic acid-rich fluid secreted by the synovial membrane, lubricates joints, nourishes avascular cartilage, and carries proteins into circulation. Changes in SF composition can indicate joint disease, such as RA [10,11,12,13]. Inflammatory cells and mediators in SF signal pathology, making it a valuable source of biomarkers. Extracellular vesicles (EVs) in SF contain exosomes and microvesicles, which may mark cartilage degradation or inflammation. Studying SF’s molecular and cellular makeup can reveal metabolic and inflammatory processes in joint diseases. A biomarker discovery pipeline includes four stages: discovery, qualification, verification, and validation (Figure 1). Proteomics identifies proteins in high-quality samples, and selected candidates undergo testing in larger sample groups. Verified biomarkers are then validated and commercialized through clinical assays approved by agencies like the European Medicines Agency (EMA) and the Food and Drug Administration (FDA) in the USA [14,15].

Proteomic analysis for arthritis biomarkers often targets biological fluids, primarily blood (serum or plasma) and SF. While the serum proteome offers a comprehensive view of internal biological activities by circulating through tissues and organs, SF, as a plasma filtrate enriched with specialized proteins, provides a more direct snapshot of joint health. Due to its proximity to critical joint structures, SF is an ideal medium for identifying arthritis biomarkers, which may also help develop serum-based tests for joint disease indicators [16]. Proteomic methods, especially mass spectrometry, enable detailed SF analysis by identifying protein profiles, expression levels, and post-translational modifications (PTMs), which can reveal disease-specific patterns [17,18]. Mass spectrometry combines non-targeted shotgun proteomics with quantitative approaches to link protein identities, concentrations, and disease states [19,20].

In RA, biomarkers can be free in SF or encapsulated within EVs. The composition of EVs depends on their origin and environment, containing elements such as citrullinated epitopes, pro-inflammatory cytokines, immune complexes, and MHC molecules. Research has shown that EVs derived from fibroblast-like synoviocytes (FLS) in RA can promote macrophage migration via PTX3 and PSMB5, rather than directly inducing cytokine release. EVs from RA SF may also contain IgG and citrullinated peptides, suggesting they could act as autoantigens in RA [21]. Exosomes from RA patients’ SF interact with citrullinated proteins—identified as autoantigens—including fibrin α-chain fragments, fibrin β-chains, fibrinogen β-chain precursors, fibrinogen D fragments, and Spα receptor, which can stimulate pro-inflammatory cytokines and immune cell activation, notably Th1 and Th17 cells [22,23].

Although many potential biomarkers for RA are under investigation (see Table 1), clinical tests remain limited to autoantibody tests (RF and anti-CCP) and inflammatory markers (ESR and CRP). These tests are constrained because they primarily detect immune responses or general inflammation, which may not fully capture early or subclinical disease states. The anti-CCP2 antibody test, though highly predictive of erosions, has limited sensitivity in the initial stages, often missing early RA cases when intervention could be the most beneficial. Expanding the biomarker pool for diagnostics and treatment is essential to improve RA patient outcomes [24]. Mass spectrometry, especially at disease sites, is particularly promising for discovering biomarkers that more accurately reflect RA’s underlying biological processes [25].

## 3. Newly Established Markers

### 3.1. Aggrecan

Aggrecan, a primary structural protein in cartilage, is broken down by metalloproteinases (MMPs) and aggrecanases, resulting in fragments that reveal neoepitopes, which indicate cartilage turnover [51]. Elevated levels of aggrecan fragments in the SF of RA patients have been linked to disease severity, making aggrecan a promising marker [52]. For instance, studies using ELISA detected significantly higher aggrecan levels in SF from patients with rapid joint destruction compared to those with slower progression [53]. Additionally, increased aggrecan fragments have been observed in patients with RA and osteoarthritis (OA), suggesting that these patients have altered aggrecan profile [54,55]. Interestingly, a unique aggrecan fragment, 374ARGSVI, may help distinguish RA from other types of arthritis, if validated, offering potential diagnostic specificity [56].

The CS846-epitope, a specific chondroitin sulfate marker detected by monoclonal antibody 846, reflects new aggrecan synthesis and is found in minimal amounts in normal cartilage. Initial studies indicated that this epitope is directly linked to aggrecan production and mirrors the breakdown of newly formed aggrecan. In early RA, the serum levels of the CS846-epitope differed among patients with varying disease progressions; those with slower joint deterioration had higher CS846 levels, while patients with rapid joint damage showed lower CS846 levels but increased C-propeptide of type II procollagen (CPII), suggesting a selective rise in collagen synthesis associated with severe cartilage turnover [57,58]. These findings underscore aggrecan’s potential as a biomarker for assessing joint damage progression and tailoring treatments in RA. Further clinical studies are needed to confirm its diagnostic and prognostic utility, paving the way for targeted therapies that could improve disease outcomes by monitoring cartilage health at a molecular level.

### 3.2. Matrix Metalloproteinases

MMPs serve as crucial biomarkers in RA due to their role in cartilage degradation [59]. In RA, inflamed synovium and activated chondrocytes produce MMPs, notably MMP-1, MMP-3, MMP-9, and MT1-MMP, which are secreted into the SF and actively degrade cartilage [59,60]. Elevated MMP-3 levels strongly correlate with joint damage and predict radiographic progression in RA patients. Rooney et al. highlighted the potential of biomarkers like MMPs in tracking therapeutic responses and predicting joint damage progression [61]. Chang et al. demonstrated significantly elevated MMP-2 and MMP-9 levels in RA, underscoring their role in disease pathogenesis [62]. Active MMPs degrade cartilage components, positioning them as both biomarkers and therapeutic targets [62,63]. However, the clinical use of MMP inhibitors remains limited due to side effects like joint stiffness and fibrosis.

Through an EV-mediated route, endothelial cells are linked to angiogenesis by activating c-Jun N-terminal kinase (JNK) signaling through the encapsulated inhibitor of DNA binding 1 (Id1) [64]. Other in vitro research has demonstrated that granulocyte- and monocyte-derived EVs induce the generation of thrombin, which has a pro-coagulatory impact. This finding raises the possibility of an EV-mediated link between coagulation and inflammation. Furthermore, it has been demonstrated that exosomes generated from FLSs include enzymes that break down the cartilage matrix and that they increase the expression of MMP-13, MMP-3, IL-6, and VEGF in chondrocytes, both of which worsen cartilage injury [65]. Together, these insights into MMP expression and activity reinforce their significant potential as biomarkers and therapeutic targets in the management of RA.

### 3.3. Glucosyl–Galactosyl–Pyridinoline

Glucosyl–galactosyl–pyridinoline (Glc-Gal-PYD) shows strong potential as a biomarker for joint destruction, particularly in RA [35]. Elevated urinary levels of Glc-Gal-PYD are linked to synovial tissue degradation and the progression of joint damage. In a study of 66 RA patients treated with infliximab and methotrexate, baseline Glc-Gal-PYD levels were significantly higher compared to the healthy controls, suggesting its utility in identifying patients at risk for rapid joint damage [66]. Glc-Gal-PYD, a glycosylated form of pyridinoline (PYD), is primarily found in synovial tissue and is released during its degradation, distinguishing it from other collagen fragments released in joint degradation. Its presence in urine correlates with joint damage severity in RA and OA, making it a promising tool for both the diagnosis and monitoring of disease progression. As a specific marker of synovial tissue breakdown, Glc-Gal-PYD may provide a more accurate measurement of disease activity, improving early diagnosis and enabling targeted treatment adjustments in joint-destructive diseases [35,37].

### 3.4. Hyaluronic Acid

Hyaluronic acid (HA) levels have attracted attention as biomarkers in RA due to their link to disease activity and joint damage. In RA patients, serum HA levels can be elevated 10- to 20-fold compared to healthy controls, with higher baseline levels correlating with long-term radiographic scores and joint damage progression [67]. However, HA levels show significant diurnal variations, peaking during physical activity, which complicates their use as consistent indicators. In early RA, elevated serum HA correlates with traditional inflammatory markers like CRP and ESR, though the associations are modest (0.436 for ESR and 0.422 for CRP) and may diminish during treatment [67]. These findings suggest the limited utility of HA as an early diagnostic tool, particularly in seronegative RA patients where elevated levels do not clearly correlate with clinical outcomes.

A study by Cylwik et al. highlighted that HA levels can vary not only among individuals with different rheumatic diseases—such as systemic sclerosis and systemic lupus erythematosus—but also within RA subgroups, emphasizing the need to account for baseline inflammation levels and disease activity in interpreting HA data [68]. Also, synovial fluid HA concentrations in RA and OA patients may be influenced by other molecular markers, such as antigenic keratan sulfate, suggesting a more complex relationship between HA levels and disease pathology [69].

### 3.5. S100 Proteins

The S100 protein family, consisting of over 20 members (e.g., S100A1–S100A18, S100B, and S100P), includes low-molecular-weight calcium-binding proteins (9000–14,000 Da) involved in calcium-dependent cellular processes. S100 proteins typically function as dimers, acting as calcium sensors to regulate gene expression, enzyme activation, cell cycle, and inflammation [70]. Their expression is notably altered in various pathological conditions, including cancer, neurodegenerative disorders, and autoimmune diseases, highlighting their potential as biomarkers [71,72]. Supplementing these findings is research on members of the S100 family in atherosclerosis or Alzheimer’s disease [73,74].

Neutrophils are a major source of S100A8, S100A9, and S100A12, with S100A8/A9 (calprotectin) comprising up to 40% of neutrophil cytoplasmic proteins, while S100A12 is present at lower levels (approximately 5–15%). Elevated levels of S100A8, S100A9, and S100A12 have been identified in the synovial fluid and serum of RA patients, highlighting their role as biomarkers for inflammation, and their potential as diagnostic and prognostic indicators of disease activity and progression [75,76].

Calprotectin, a member of the S100 family, is a valuable biomarker for diagnosing inflammatory conditions, including RA [77]. It is a heterodimer of two calcium-binding proteins, MRP-8 (S100A8) and MRP-14 (S100A9), found in granulocytes and monocytes [78]. At inflammation sites, calprotectin is released as an acute-phase reactant, with levels increasing significantly, often over 100-fold during active inflammation [77]. Unlike CRP and ESR, which are produced in the liver, calprotectin is produced directly at the inflamed synovial tissue, providing a more localized measure of inflammation [79,80]. This unique characteristic makes calprotectin particularly useful for assessing RA activity and progression, especially alongside other S100 family proteins, like S100A12 and S100A4, which are also linked to chronic inflammatory states.

### 3.6. Cytokines/Inhibitors/Adipocytokines

In RA, an imbalance between pro- and anti-inflammatory cytokines drives the chronic inflammatory processes that characterize the disease [81]. Key pro-inflammatory cytokines, including IL-1, IL-6, IL-15, IL-17, TNF-α, and granulocyte macrophage–colony stimulating factor (GM-CSF), are elevated in the synovial fluid and serum of RA patients, contributing to synovial inflammation, cartilage destruction, and bone erosion [45]. IL-6, one of the most abundant cytokines in RA, plays a central role in systemic inflammation and correlates with disease activity. Therapeutic targeting of the IL-6 receptor has demonstrated significant clinical benefits, though the direct link between IL-6 levels and joint damage remains an area of ongoing study [82].

In addition, markers like soluble TNF receptor II (sTNFR-II) have shown promise as early indicators of RA, with elevated levels detectable years before clinical onset, highlighting their potential role in preclinical diagnosis [83]. In early synovitis cases that progress to RA, unique cytokine profiles have been identified. These include elevated levels of IL-2, IL-4, IL-13, IL-15, and IL-17, which promote inflammatory responses and osteoclastogenesis. Among these, IL-17 is particularly potent in driving bone resorption and joint destruction, making it a key target for therapeutic intervention [46,47].

Chemokines such as CXCL8 (IL-8) and CCL2 (MCP-1) are significantly elevated in RA synovial fluid and correlate with disease activity [84]. Additionally, CXCL13, a chemokine associated with B-cell trafficking, and CCL23, which recruits monocytes and other immune cells, have been identified as potential biomarkers of disease activity in RA patients [85]. Despite these findings, traditional markers like ESR and CRP remain the most reliable for clinical RA assessment.

### 3.7. Immunological Markers

T-cell infiltrates are a key indicator of a high concentration of CD4+ T cells, particularly regulatory T cells (Tregs) like CD4 + CD25+ Tregs, which are vital for maintaining self-tolerance and preventing autoimmunity [86]. However, Tregs may function inadequately or be defective in RA [87,88]. Studies have shown that while CD4 + CD25+ Tregs are abundant in inflammatory joints and have strong suppressive effects, RA progression continues, possibly due to the reduced sensitivity of effector cells to Treg suppression [49,89]. Van Amelsfort et al. demonstrated elevated Treg levels in synovial fluid compared to peripheral blood, but their suppressive function was significantly diminished in the inflammatory environment of RA [89]. Additionally, proinflammatory cytokines, such as TNF-α and IL-6, have been shown to interfere with Treg-mediated suppression, further contributing to chronic inflammation and joint damage [50]. While Tregs hold potential as biomarkers for immune regulation in RA, findings regarding their diagnostic utility remain inconsistent [49,88].

The current RA biomarker research includes markers such as CTX-I, CTX-II, COMP, Glc-Gal-PYD, MMP3, and C2C in the urine and serum. Although correlations between these markers and patient outcomes have been noted, their interactions and added value over traditional markers remain unclear. A combined biomarker panel could improve prognosis and aid in identifying patients likely to experience aggressive disease, enabling more personalized treatment decisions and potentially sparing others from intensive therapies.

## 4. Novel Markers

### 4.1. ADAMEDC1

RA and OA share similar symptoms but differ fundamentally in their underlying mechanisms. Analyzing differentially expressed genes (DEGs) between RA and OA could significantly enhance diagnostic precision and inform targeted treatments. In the study by Kang et al., RA samples showed the upregulation of 14 genes and the downregulation of 3 compared to OA, with gene ontology analysis indicating these DEGs largely regulate inflammation in synovial tissue. Notably, ADAMDEC1 in SF emerged as a promising RA biomarker, potentially aiding in early molecular diagnosis and targeted therapies [90]. The current diagnostic methods for RA and OA, such as imaging and symptom evaluation, are limited in detecting early disease stages, as they primarily assess structural changes and physical function [91]. DEGs like CXCL13, CD247, GZMB, CCL18, IL7R, and ADAMDEC1, identified by Kang et al. and validated by Li et al., reveal molecular distinctions between RA and OA, offering more precise diagnostic and therapeutic targets [92].

The validation of ADAMDEC1 in clinical SF samples demonstrated its robustness in identifying RA lesions, even in patients’ post-joint replacement, underscoring its potential as a diagnostic and prognostic biomarker. The limitations of Kang’s study included a small cohort size and advanced disease stages among the participants. Future research should explore ADAMDEC1’s efficacy in detecting early-stage RA, aiming to establish its role in guiding early intervention and personalized treatment [90].

### 4.2. MAGE-I-mRNA

A study by Aiman et al. evaluated melanoma-associated antigen gene 1 (MAGE-1) mRNA expression in SF cells, alongside the serum levels of anti-CCP and rheumatoid factor (RF) as early diagnostic biomarkers for RA [93]. The study included 213 participants, with 135 RA patients and 78 patients with traumatic knee injuries as controls. Using ELISA for RF and anti-CCP and RT-PCR for MAGE-1 mRNA, the researchers found significantly elevated serum RF IgM and anti-CCP levels in the RA patients compared to the controls, showing a strong correlation between the two. Remarkably, MAGE-1 mRNA expression was present in 100% of the RA patients but absent in the controls, yielding 100% sensitivity and specificity for MAGE-1, RF, and anti-CCP as diagnostic biomarkers.

The study concluded that MAGE-1 expression in SF cells holds promise as a specific and sensitive diagnostic biomarker for RA. The researchers advocate combining MAGE-1 transcript analysis with RF and anti-CCP to enhance early RA detection, which could lead to improved patient management and outcomes. Further studies with larger cohorts are recommended to confirm MAGE-1’s predictive value and role in chronic RA inflammation. The research underscored the established values of RF and anti-CCP while introducing MAGE-1 as a potential addition to the diagnostic toolkit for early RA intervention [94].

### 4.3. Non-Coding RNAs

Murata et al. explored microRNAs (miRNAs) in SF and plasma as potential biomarkers for distinguishing RA and OA. Their study assessed specific miRNAs (miR-16, miR-132, miR-146a, miR-155, and miR-223) in the SF of RA and OA patients and in the plasma of RA, OA, and healthy individuals. Using quantitative PCR, they found that miRNA expression differed markedly between SF and plasma, indicating that SF miRNAs likely originate from synovial tissue. Plasma miR-132 successfully differentiated RA and OA patients from the healthy controls, while the SF miRNAs were particularly useful in distinguishing RA from OA. The plasma miRNAs were also correlated positively with RA disease activity, making them valuable for clinical assessments. Notably, miR-16, miR-146a, miR-155, and miR-223 were elevated in RA, underscoring their diagnostic potential [95].

Recent studies have aimed to investigate exosomal miRNAs and other arthritis-related illnesses. Exosomes in synovial fluid are closely linked to arthritis pathophysiology, contributing to joint deterioration, inflammation, and cartilage degradation [22]. Through the exchange of lipids, proteins, various signaling molecules, and nucleic acids—including miRNAs and long non-coding RNAs (lncRNAs)—exosomes are involved in various pathological and physiological processes [96]. miRNAs are the most extensively researched compounds in exosomes and have been employed as potential biomarkers [97,98]. Xie et al. isolated exosomes from osteoarthritis patients’ serum and synovial fluid, using miRNA sequencing to examine the expression. They identified 31 upregulated and 33 downregulated miRNAs in synovial fluid compared to serum, suggesting that serum-derived exosomes may not accurately represent synovial fluid exosomes. These differentially expressed miRNAs were mostly linked to metabolic and cancer-related pathways, providing insights into joint conditions and early indicators for arthritis diagnosis [99]. Notably, miRNAs such as miR-155, miR-150, miR-146a, and miR-21 are often elevated in exosomes from RA patients and are related to IL-17-producing T cell differentiation in RA patients [100,101]. miR-146a, a miRNA that inhibits osteoclasts from destroying bone, and miR-221-3p, an FLS-derived miRNA involved in bone signaling pathways, have been suggested as RA diagnostic biomarkers [102,103]. Yu et al. studied the function of two exosomal miRNAs, miR-335p, and miR-483-5p. They discovered that the SRSF4 gene, which is in charge of cartilage repair, is a typical target for these sequences. This gene’s expression was downregulated, which resulted in the inhibition of cartilage regeneration [104].

Additionally, the homeostasis and imbalance of T-regulatory cells (Tregs)/Th cells have also been connected to exosomes and their miRNA. In particular, the downregulation of TGFBR II (transforming growth factor beta receptor II) expression was linked to the overexpression of miR-17, preventing Treg development in vitro [105].

Additionally, lncRNAs in SF exosomes, particularly a specific transcript—ENST00000433825.1—strongly correlated with CRP levels, which are traditional inflammatory markers. This unique lncRNA expression profile in RA SF may aid in differentiating RA from other joint diseases, such as OA and gout, and offers diagnostic and therapeutic insights [106].

### 4.4. GFAP and A1BG

Biswas et al. used an immune-proteomic approach to identify novel autoantigens involved in RA pathogenesis, analyzing SF samples from RA patients with non-RA rheumatic samples (OA and trauma) as controls. Through two-dimensional gel electrophoresis (2-DE) and Western blotting, they identified 18 distinct autoantigens in RA, including five key proteins: vimentin, gelsolin, alpha 2 HS glycoprotein (AHSG), glial fibrillary acidic protein (GFAP), and a1B-glycoprotein (A1BG), which showed significantly higher expression in RA SF than in the controls [107]. GFAP has been previously discovered as a novel biomarker of an autoimmune disorder [108]. A1BG is a human plasma protein with unknown functions [109]. ELISA tests further confirmed elevated autoantibodies against GFAP and A1BG in RA plasma compared to OA patients, suggesting their potential as diagnostic biomarkers [107].

The aforementioned study highlights GFAP and A1BG as promising new biomarkers for RA, addressing the limitations in the current RA diagnostics that often rely on non-specific antibody assays. Identifying these autoantigens could improve early diagnosis and prognosis, enabling more personalized treatment approaches. These findings demonstrate the utility of immunoproteomic methods in autoimmune research and suggest similar strategies could be applied to other autoimmune conditions.

### 4.5. ORM2

Kim et al. investigated orosomucoid-2 (ORM2), an acute-phase protein, in RA, revealing its substantial role in chronic inflammation. Their proteome profiling showed that ORM2 was significantly upregulated in the serum of RA patients, displaying the highest fold change among acute-phase reactants [110]. ORM2 was also elevated in RA synovial fluid and membranes, primarily produced by synovial macrophages and FLSs. The study demonstrated that recombinant ORM2 stimulates RA macrophages and FLSs to produce proinflammatory cytokines (IL-6 and TNF-α) and chemokines (CCL2 and CXCL8) via the NF-κB and p38 MAPK signaling pathways. Glycophorin C was identified as a receptor mediating ORM2’s proinflammatory effects [111].

In mouse models, intra-articular ORM2 injections increased arthritis severity and promoted macrophage infiltration. Serum ORM2 levels were correlated with RA activity and radiographic progression, highlighting its potential as a biomarker for assessing disease severity. The study suggested that ORM2 triggers reciprocal activation between macrophages and FLSs, creating a feedback loop that intensifies inflammation. While limited by short experimental durations and a lack of ORM2-neutralizing antibodies, the research established ORM2 as a promising biomarker and therapeutic target in RA, with liver-derived ORM2 potentially contributing to RA pathogenesis [111].

### 4.6. 14-3-3η Protein

Hammam et al. studied the 14-3-3η protein as a potential biomarker for assessing joint damage and predicting radiographic progression in established RA. While traditional markers like RF and anti-CCP are commonly used, their effectiveness is limited by issues such as seronegativity, impacting their predictive value for joint damage [112,113]. The study highlighted the need for biomarkers associated with joint damage mechanisms to improve radiographic predictions and inform management strategies [114].

The researchers found that elevated serum and SF levels of 14-3-3η were significantly correlated with radiographic damage and erosion scores. Notably, the SF levels of 14-3-3η were significantly higher than the serum levels, underscoring its value as a direct indicator of joint damage processes [115]. The protein was shown to stimulate pro-inflammatory cytokines, including TNF-α and IL-6, as well as matrix metalloproteinases (MMPs), which contribute to cartilage and bone destruction [115,116]. Additionally, the correlation between SF 14-3-3η levels and patient age suggests that older RA patients, who are at greater risk of severe joint deterioration, may benefit from therapies tailored to their 14-3-3η profile [117].

See Table 2 for the categorization of biomarkers.

## 5. Specific Applications of Extracellular Vesicles

**Figure 2:**
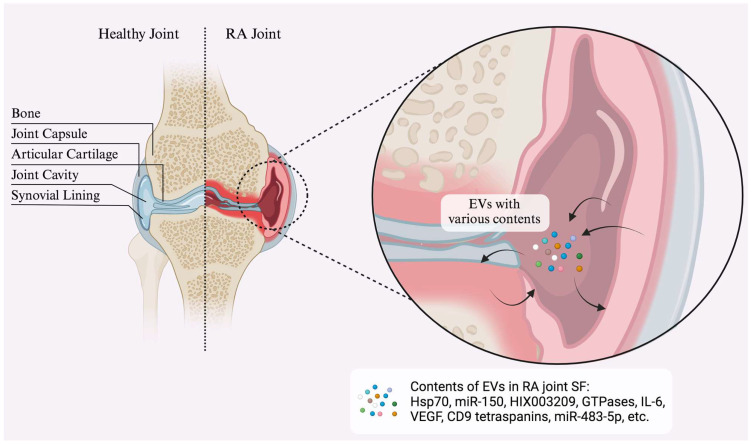
Healthy joint vs. joint affected by RA.

Exosomes, a specific type of EVs between 40 and 100 nm in size, play a vital role in cellular communication by transporting molecular cargo among cells. In RA, exosomes are abundant in synovial fluid and primarily derived from platelets, as well as monocytes, neutrophils, and granulocytes. Both B and T cells also produce exosomes in the synovial fluid, with the quantity of T-cell-derived exosomes correlating strongly with blood rheumatoid factor levels [118,119]. See Figure 2 for the exosomal contents in an RA joint.

By mediating joint destruction and promoting pathological processes such as cartilage degradation and bone erosion, exosomes contribute significantly to disease progression in RA. Specifically, exosomes derived from RA FLS can stimulate CD4+ T cells, augment IFNγ and IL-2 production, amplify NF-κB and Akt activation, and prevent caspase 3 and caspase 8 cleavages [120]. These exosomes can break down bone and cartilage through direct metalloproteinase activity or the autocrine activation of MMP-1 released by FLS. Additionally, FLS can be the target of exosomes produced by monocytes and activated T cells, leading to an increased release of MMPs, cytokines, and chemokines, and potentially resulting in angiogenesis and the chemokine-induced migration of human microvascular endothelial cells [121]. Exosomes also promote osteoclast activity and bone resorption by upregulating RANK and RANKL expression, critical factors in bone metabolism and RA-associated bone erosion [122]. Given their impact on inflammation, cartilage degradation, and bone resorption, exosomes represent promising targets for diagnostic and therapeutic strategies in RA. The contents of exosomes are summarized in Table 3.

### 5.1. Heat Shock Proteins

In RA, transmembrane exosomal markers, such as chaperones Hsp70 and Hsp90 (internal components) and tetraspanins CD9 and CD81 (surface markers), are significantly elevated, with increases of 3.2-fold for CD9 and 2.2-fold for Hsp70. These markers serve as potential indicators of exosomal involvement in RA, contributing to inflammation and immune response regulation. Specifically, CD9 and Hsp70 have shown promise as diagnostic markers due to their strong expression in RA-related exosomes. Exosomal proteins also exhibit bidirectional changes in enzymatic activity in RA. Acidic endonucleases, responsible for DNA and RNA breakdown, show a dramatic increase in activity (up to 55–30-fold), while alkaline endonucleases decrease by 2–3 times. This altered enzymatic activity reflects the acidic shift in SF pH seen in RA, from a normal 7.75 to 7.21, which influences the exosomal secretion of proteins and nucleic acids. This pH-dependent change affects intercellular immunomodulatory communication, suggesting that exosomes not only serve as biomarkers but could also be targeted to modulate inflammation [123].

### 5.2. Lysophosphatidylcholine, Sphingolipids, and Cholesterol

Lipid molecules, including lysophosphatidylcholine, sphingolipids, and cholesterol, play emerging roles in RA-associated inflammation. During acute inflammation, phosphatidylethanolamines and hexosylceramides increase significantly, suggesting a potential link between lipid changes and synovial inflammation. Specific lipids, such as fatty acids, metabolites, and lipoproteins, exhibit immunomodulatory and anti-inflammatory effects, which could impact RA progression. Additionally, lipid classes like hexosylceramides and sphingomyelin are involved in EV biogenesis, potentially influencing EV-mediated communication in RA. However, understanding the role of lipid content within EVs in RA is still in the early stages, and further research is needed to determine their precise impact on disease mechanisms [124].

### 5.3. Proteomic Analysis of EVs

Proteomic analysis of EVs in the synovial fluid of RA patients has revealed a range of pro-inflammatory proteins that may drive disease progression. Key factors include signal transducers like STAT1 and STAT3, tyrosine kinases JAK1, JAK2, and TYK2, TLR2, and apoptosis-associated PYCARD. Additionally, MMP9, PRKCB, CCR1, leukotriene A–4 hydrolase (LTA4H), and several S100 calcium-binding proteins were identified, indicating their roles in joint inflammation and cartilage breakdown. Autoantigen-associated proteins, such as calreticulin-3, vimentin, HSP90, and histones H1, H2, and H3 were also present, suggesting EVs’ role in autoantigen presentation in RA [125]. Moreover, elevated levels of GTPases (KRas, NRas, Ral-A, and RhoA) and components of the NADPH oxidase complex (RAC1, RAC2, CYBA, and CYBB) indicate involvement in immune response regulation [65].

Further research by Yoo et al. explored serum exosomes from 60 RA patients, half in clinical remission and half with active disease. The results show that arachidonic acid (AA) levels were higher in patients with active disease, while LYVE-1 levels were lower, suggesting these markers could aid in assessing RA disease activity and progression [126].

## 6. Conclusions

The study of biomarkers in RA, especially those derived from synovial fluid and EVs, opens new avenues for understanding disease mechanisms and enhancing clinical care. Synovial fluid biomarkers provide direct insight into joint-specific inflammatory and degenerative processes, offering a more precise assessment of the disease state than systemic markers like serum proteins. EVs, which carry proteins, lipids, and genetic material, such as microRNAs and heat shock proteins, are particularly promising due to their roles in modulating immune responses and inflammation in RA. These vesicles act as biomarkers and carriers of molecular signatures that reflect disease activity and progression.

As research advances, these biomarkers hold significant promise for improving RA diagnosis, tracking disease activity, predicting therapeutic responses, and identifying patients at higher risk for joint damage. Enhanced the understanding and application of these markers could lead to more personalized, effective RA management strategies and improved patient outcomes.

## Figures and Tables

**Table 1:** The categorization of RA markers and their locations and potential associations with the disease.

Marker Type	Marker Name/Description	Location	Association with RA	Reference
Genetic Markers	HLA-DRB1	Genetic–6p21.32	The strongest association with RA risk and severity, also with anti-CCP antibodies.	[26]
	TRAF1/C5	Genetic–9q33-34	Associated with erosive changes in RA, though of modest clinical utility.	[27]
	PADI4	Genetic–1p36.13	Controversial association with RA susceptibility across different populations.	[28]
Bone Markers	RANKL/OPG ratio	Serum/synovium	Predicts radiographic progression of joint damage.	[29]
	Collagen (type I) cross-linked C-telopeptide (CTX-I)	Serum/urine	High levels predict the risk of radiological progression and joint destruction.	[30]
	Bone sialoprotein (BSP)	Serum/synovial fluid	Elevated levels may indicate joint damage and help in the early identification of destructive RA.	[31]
	Cathepsin K	Serum/synovial fluid	Potential marker of bone resorption but requires further validation.	[32]
	Osteocalcin	Serum	Reflects bone metabolism but is less effective at predicting disease activity.	[33]
Cartilage Markers	Cartilage oligomeric matrix protein (COMP)	Serum	Changes in COMP linked to cartilage destruction and joint damage in RA.	[34]
	C-terminal cross-linking telopeptide of type II collagen (CTX-II)	Urine	High levels predict joint destruction progression in RA.	[30,35]
	Col2-3/4Clong mono and Col2-3/4Cshort (Type II)	Serum	May indicate cartilage degradation and predict joint space narrowing.	[36]
	Hyaluronic acid (HA)	Serum/synovial fluid	Elevated levels correlate with disease activity but are variable throughout the day.	[35,37]
Autoantibodies	Rheumatoid factor (RF)	Serum	Commonly detected but not specific to RA; correlates with disease activity and prognosis.	[38]
	Anti-cyclic citrullinated peptide (anti-CCP)	Serum	Highly specific for RA and predictive of joint destruction; may be detected before RA onset.	[39]
	Anti-mutated citrullinated vimentin (MCV)	Serum	High specificity for RA; predicts severe joint involvement.	[40]
	Antiperinuclear factor (APF)	Serum	Present in many RA patients; assists in early intervention.	[41]
Inflammatory Markers	Erythrocyte sedimentation rate (ESR)	Blood	Reflects disease activity and can predict long-term radiological progression useful in clinical settings.	[42]
	C-reactive protein (CRP)	Blood	Reflects short-term disease activity and is associated with joint destruction; widely used.	[32]
	Calprotectin	Synovial fluid	High levels correlate with joint damage and predict erosive disease.	[43]
	Serum amyloid-associated protein (SAA)	Blood	Correlates with clinical parameters; may not provide additional information over CRP.	[44]
Cytokines, Inhibitors Adipokines	Various cytokines (e.g., IL-1, IL-6, TNF-α)	Synovial fluid	Elevated levels contribute to joint destruction; dynamic balance is necessary for predictive value.	[45,46]
	Soluble TNF receptor II (sTNFR-II)	Blood/synovial fluid	May indicate future RA development.	[47]
	Adipocytokines (e.g., leptin, visfatin)	Blood	Elevated in RA; associated with radiographic joint damage.	[48]
Immunological Markers	CD4 + CD25+ T-regulatory cells	Synovial fluid	Increased in inflamed joints; controversial role in RA pathology and potential biomarkers.	[49,50]

**Table 2:** Categorization of discussed biomarkers.

Marker	Prognostic	Diagnostic	Therapeutic
Aggrecan	+	+	−
MMPs	+	−	+
Glc-Gal-PYD	+	−	−
Hyaluronic acid	−	+	−
S100 proteins	−	+	−
Calprotectin	−	+	−
Cytokines	+	−	+
Immunological markers	−	−	−
ADAMEDC1	+	+	−
MAGE-I-mRNA	−	+	−
Non-coding RNAs	+	+	+
GFAP and A1BG	+	+	−
ORM2	+	−	+
14-3-3η protein	+	+	−

**Table 3:** The contents of exosomes and their roles in RA.

Exosomal Content	Source/Type	Role in RA	Key Markers/Findings	Ref.
miRNAs	Synovial fluid exosomes	Modulate inflammation, serve as biomarkers, contribute to joint damage and cartilage degradation	miR-155, miR-150, miR-146a, miR-21, miR-221-3p, miR-335p, miR-483-5p; associated with T cell differentiation and inhibition of bone repair	[97,98]
Autoantigens	EVs from fibroblast-like synoviocytes (FLS)	Participate in the immune response by interacting with citrullinated proteins and presenting autoantigens	Citrullinated peptides, MHC-I/II molecules, immunological complexes, IgG, fibrin α-chain, β-chain, fibrinogen	[20]
Heat Shock Proteins (HSPs)	Exosomes from synovial cells	Contribute to inflammatory pathways and exosome secretion	Elevated Hsp70 and CD9 tetraspanins; increased acidic endonuclease activity and decreased alkaline activity	[123]
MMPs	Exosomes from FLS	Promote cartilage breakdown and angiogenesis	MMP-13, MMP-3, IL-6, VEGF; granulocyte-derived vesicles linked to pro-coagulation effects	[65]
Lipids	Exosomal lipid content	Involved in synovial inflammation and EV biogenesis	Hexosylceramides, sphingomyelin; increased phosphatidylethanolamines during acute inflammation	[124]
Proteins	EVs in synovial fluid	Associated with inflammation, bone destruction, autoantigen presentation, and signaling pathways	STAT1, STAT3, JAK1, JAK2, TLR2, MMP9, PYCARD, CCR1, HSP90AB1, F-actin, Histones H1, H2, H3; GTPases, NADPH oxidase, MPO, Rac1, Rac2	[125,126]
lncRNAs	Exosomal lncRNAs	Serve as RA biomarkers; modulate inflammation via miRNA interaction	ENST00000433825.1 (correlates with CRP levels in RA); HIX003209 linked to inflammatory processes	[106]

## Data Availability

Not applicable.
